# Ecological risk and health risk analysis of soil potentially toxic elements from oil production plants in central China

**DOI:** 10.1038/s41598-022-21629-y

**Published:** 2022-10-12

**Authors:** Lu Gan, Jiangping Wang, Mengyun Xie, Bokai Yang

**Affiliations:** 1 School of Urban Design, Wuhan University, Wuhan, 430072 Hubei China; 2 College of Art, Yangtze University, Jingzhou, 434023 Hubei China; 3 College of Art, Minnan Normal University, Zhangzhou, 363000 Fujian China

**Keywords:** Ecology, Environmental sciences

## Abstract

In this study, the enrichment factor (EF) and pollution load index (PLI) were used to evaluate the pollution of potential toxic elements (PTEs) in the soil near the oil production plants in central China, and the potential ecological risk (PER) and human health risk (HHR) assessment model were used to evaluate the PER and HHR caused by the soil PTEs in the study area. The mean EFs of all PTEs were greater than 1, PTEs have accumulated to varying degrees, especially Cr, Ni and Pb were the most serious. The average value of PLI was 2.62, indicating that the soil PTEs were seriously polluted. The average ${E}_{r}^{i}$ values of PTEs were Cr > Pb > Cd > Ni > As > Cu > Zn > Mn, of which Cr, Pb, Cd and Ni were at medium and above PER levels. Both adults and children in the study area suffered from varying degrees of non-carcinogenic and carcinogenic risks. The total hazard index (THI) values of children (7.31) and adults (1.03) were all > 1, and the total carcinogenic risk index (TCRI) of children (9.44E−04) and adults (5.75E−04) were also > 10^–4^. In particular, the hazardous quotient (HQ) of Cr and Pb for children under the oral intake route were 4.91 and 1.17, respectively, caused serious non-carcinogenic risk. And the carcinogenic risk index (CRI) values of the PTEs in adults and children under the three exposure routes were Cr > Ni >  > As > Pb >  > Cd. Among them, the CRI values of Cr and Ni in children and adults by oral intake were both greater than 10^–4^, showing a strong carcinogenic risk. The results will provide scientific basis for environmental protection and population health protection in this area.

## Introduction

The rapid development of industrialization and urbanization has promoted the remarkable development of economy, and also caused the destruction of soil ecological environment, especially the problem of soil potential toxic element (PTEs) pollution caused by excessive enrichment of PTEs in the soil^1–4^. In addition, PTEs were highly toxic, difficult to degrade, and easy to accumulate^5^. After entering the ecosystem, they were prone to migrate and accumulate, destroy the ecological balance, and affect human health through the food chain, causing widespread concern in the world^5–7^. Soil PTEs mainly include natural and man-made sources, among which man-made sources are mainly related to metal mining, metal smelting and processing, automobile exhaust emissions, and oil well extraction activities and so on^8–10^. The investigation found that there were few reports on the pollution assessment and risk assessment of the soil PTE around oil production plants. Crude oil is generally rich in various PTEs, and in the process of oil well extraction, oil and gas exploitation, oil and gas transportation, and oil well maintenance, etc. can cause crude oil to spill, leading to the accumulation of PTEs in the soil around oil production plants^11–13^. When the accumulation of PTEs in the soil exceeds the safety threshold range, the ecological balance and human health will be threatened^14^. Therefore, it is of great significance to deepen the pollution assessment and risk assessment of the soil PTEs around oil production plants.

The potential ecological risk (PER) model comprehensively considers the toxicity level of PTEs, the environmental background value and the synergy of each PTEs, and uses the toxicity response coefficient and the corresponding background value to comprehensively evaluate the ecological risk of PTEs. This method is widely used to evaluate the PER caused by PTEs in soil and dust^13–15^. The human health risk (HHR) assessment model proposed by the U.S. Environmental Protection Agency (US EPA) was used to evaluate the health risks of adults and children^16^. The HHR model calculates the average daily exposure of PTEs under the three routes of oral intake, oral and nose inhalation and direct skin contact, and combines it with the corresponding reference dose (RfD) and slope factor (SF) to quantify the non-carcinogenic risk and carcinogenic risk of children and adults caused by PTEs^17–19^. The HHR model quantifies the non-carcinogenic risks and carcinogenic risks that each PTE may cause, and more intuitively show the contribution of each PTE to human health risk, so as to determine the main contributing elements that lead to the carcinogenic risk and the non-carcinogenic risk^20, 21^. Therefore, prioritizing the management and control of metal smelting, processing, agricultural activities, traffic emissions, oil and gas extraction and other activities that will produce major contributing elements can effectively reduce human health risks caused by excessive accumulation of soil PTEs^22, 23^.

The study area is in the central and southern Hubei Province, central China. The area is rich in mineral resources, the main minerals are petroleum, coal, pyrite, limestone, etc., and the transportation is very convenient. Abundant mineral resources and convenient transportation have promoted the development of the local mining industry. In recent years, the petroleum industry in the study area has developed rapidly, which has put tremendous pressure on the local ecological environment. Crude oil leakage during oil well production and crude oil dripping from equipment such as oil and gas buffer tanks, oil–water pump houses and oil storage tanks have caused PTEs in crude oil to accumulate, migrate and transform in the soil near the oil well, threatening local ecological safety and human health. Thus far, few studies have systematically investigated and described the pollution characteristics, spatial distribution, potential ecological risks and human health risks of soil PTEs near the oil production plants.

Therefore, this study takes soils in the oil production plants as the research object. The main objectives of the study are to (1) investigate the accumulation and pollution characteristics of soil PTEs in the oil production plants; (2) study the spatial distribution trend of the PTEs content in the soil through geostatistics; (3) assess the ecological risks and human health risks caused by soil PTE in the study area applying PER and HHR models.

## Materials and methods

### Study area

**Figure 1:**
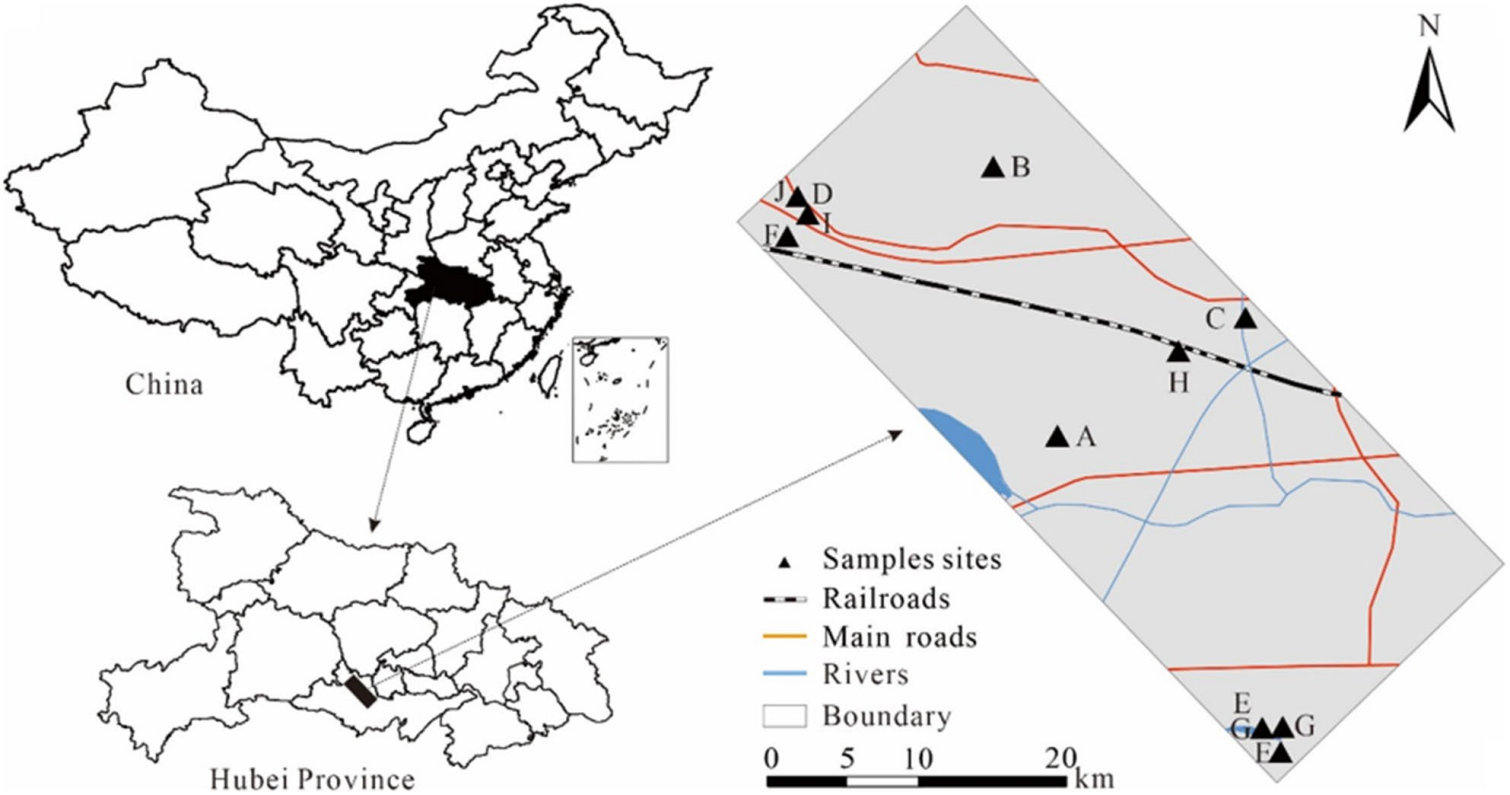
Sampling point map of the study area.

The study area is situated at the central and southern Hubei Province, central China (Fig. 1). It has a subtropical monsoon climate, with annual mean precipitation and temperature of 1100–1300 mm and 15.9–16.6 ℃, respectively. The mineral resources, including petroleum, coal, etc., are rich in the study area. In addition, the area is accelerating the construction of a modern comprehensive transportation hub integrating "iron, highway, water and air", which will become the important logistics channels of Yangtze River Economic Belt.

### Sampling and analytical methods

A total of 20 surface soil samples (0–20 cm in depth) were collected in the study area in June 2021 (Fig. 1). At each site of sampling, 3–5 subsamples were collected to form a composite sample which was stored in a polyethylene zip-bag, and was immediately transported to the laboratory.

After being air-dried, ground and sieved with a 1 mm stainless-steel mesh, soil samples (each 0.1 g) were digested with concentrated acid mixtures of HNO_3_-HF-HClO_4_. All digested solutions were filtered and diluted with ultrapure water to appropriate contents for instrumental analysis. The contents of As, Cd, Cr, Cu, Mn, Ni, Pb, Zn and Ba in all samples were analyzed using an ICP-MS. To control quality, certified reference materials including GBW07424, and GSS-24 were used to estimate the recoveries of element analysis, and the recoveries for all PTEs were in the range of 81%-109%. In addition, 20% samples were randomly selected for repeated testing, and the error was kept within 5%.

### Enrichment factor

Enrichment factor (EF) is an important indicator for quantitative evaluation of pollution degree of soil PTEs^24–26^. Reference elements with stable chemical properties were often selected as reference standards, and the elements in the test samples are normalized to reduce human influence during sampling and sample preparation and to ensure the comparability and equivalence of various indicators. Mn, Al, Ti, Sc, Zr, etc., are the commonly used reference elements^14, 27^. This paper chose Mn as the reference element when calculating the enrichment factor, and the calculation formula is as follows:

1 $$EF=\frac{{{(C}_{i}/{B}_{r})}_{sample}}{{{(C}_{i}/{B}_{r})}_{background}}$$

Here, ${C}_{i}$ was the content of PTE, $and$ ${B}_{r}$ represent the content of reference element. The degree of enrichment of PTEs were divided into 6 levels, namely non-enriched (EF value < 1), mild (1–2), moderate (2–5), significant (5–20), high (20–40) and extremely enriched (EF value ≥ 40)^24^.

### Pollution load index

Pollution Load Index (PLI) can express the contribution degree of each PTE to pollution^28, 29^. The calculation formula is:

2 $$CF=\frac{{C}_{i}}{{C}_{b}}$$

3 $$PLI=\sqrt[n]{{CF}_{1}\times {CF}_{2}\times {CF}_{3}\cdots \cdots \times {CF}_{n}}$$

where, contamination factor (CF) represents the pollution level of a single element, ${C}_{i}$ and ${C}_{b}$ were the contents of PTE to be tested and its background value, and the soil background value of Hubei province was utilized for this study. Classify PLI, 0 < PLI ≤ 1, 1 < PLI ≤ 2, 2 < PLI ≤ 3, 3 < PLI ≤ 4, 4 < PLI ≤ 5 and PLI ≥ 5 respectively correspond to no, moderate, medium high, relatively high, high and extremely high pollution^14^.

### Potential ecological risk model

Potential ecological risk (PER) combines the concentration of PTEs with their ecological and environmental effects, and toxicological effects to evaluate the potential risks of various PTE pollutants in sediments and soils to organisms. The calculation formula is as follows^23^:

4 $$\mathrm{PER}=\sum_{i=1}^{n}{E}_{{\varvec{r}}}^{i}$$

5 $${E}_{r}^{i}={T}_{r}^{i}\times CF$$

Here, ${E}_{{\varvec{r}}}^{i}\mathrm{ and}\boldsymbol{ }{T}_{r}^{i}$ represented single ecological risk for an PTE and toxic response factor of PTE, respectively. The Hakanson classification standard was based on the toxicity coefficients of PCB, Cd, Hg, Pb, As, Cr, Zn and Cu, which were different from the PTE types in this study^30^. In order to make the PER assessment results more accurate, this study adjusted the ecological risk classification standards. Hakanson potential ecological risk method classifies ${E}_{{\varvec{r}}}^{i}$ and PER according to the maximum toxicity coefficient and the sum of toxicity coefficient of 8 pollutants. First, the classification value of the unit toxicity coefficient was PERu = 150/133 = 1.13, where 150 was the primary classification limit of PER, and 133 was the total toxicity coefficient of the 8 pollutants in the original method. The PER with the largest toxicity coefficient in this study was Cd (30). The total toxicity coefficient of PTEs in this study was 59. After adjustment, the first-level limit was L1 = 59 × 1.13 ≈ 70, and the remaining limits were in turn of the upper limit double. The ${E}_{{\varvec{r}}}^{i}$ and PER grading standards before and after adjustment in this study were shown in Table S1.

### Human health risk model

The health risks of soil PTEs to the human body included non-carcinogenic risk and carcinogenic risk^31, 32^. And oral intake, oral and nose inhalation and direct skin contact were the main routes leading to human health risks. The daily average exposure of PTEs in these three exposure routes was calculated as follows^23^:

6 $${ADD}_{{jk}_{ing}} =\frac{{C}_{jk}\times IngR\times EF\times ED}{BW\times AT}\times {10}^{-6}$$

7 $${ADD}_{{jk}_{inh}} =\frac{{C}_{jk}\times InhR\times EF\times ED}{PEF\times BW\times AT}$$

8 $${ADD}_{{jk}_{der}} =\frac{{C}_{jk}\times SA\times AF\times ABS\times EF\times ED}{BW\times AT}\times {10}^{-6}$$

Here, ${C}_{jk}$ was the content of PTE *k* in the sample *j*, and the parameter values of formula (6)–(8) were exhibited in Tables S2. HQ_i_ was used to indicate the non-carcinogenic risk of PTE under different exposure routes, and it was defined by the quotient of ADD for each PTE and the corresponding reference dose. The total hazard index (THI) is reckoned as follow:

9 $$HI=\sum {HQ}_{{jk}_{i}}=\sum \frac{{ADD}_{{jk}_{i}}}{{RfD}_{i}}$$

10 $$THI=\sum HI$$

Similarly, CR was used to assess the carcinogenic risk of humans exposed to PTE and was defined by the product of each PTE’s ADD and its corresponding slope factor. The total carcinogenic risk index (TCRI) was shown in formulas (11) and (12):

11 $$CR=\sum {ADD}_{{jk}_{i}}\times {SF}_{i}$$

12 $$TCRI=\sum CR$$

The parameters of formula (9)–(12) are shown in Table S3.

## Results and discussion

### Description of PTEs

The descriptive statistics of the contents of soil PTEs in the study area were shown in Table 1. From Table 1, the mean contents of As and Ni in the oil-affected soils exceeded their corresponding risk screening values^33^, which may damage the soil ecological environment and affect crop growth. Compared with the secondary standard of soil environmental quality^34^, the mean contents of As, Cu and Zn were all lower than their corresponding Grade II standard values, but the mean contents of Cd, Cr, Ni and Pb in the oil-affected soils were 1.07, 7.46, 7.14 and 1.36 times of their standard values. In contrast with the background value of Hubei province^35^, except Mn, the mean contents of As, Cd, Cr, Cu, Ni, Pb, Zn and Ba in the oil-affected soils all exceeded their background values. Meanwhile, the variation coefficient of Cr (1.41) was greater than 1. In general, the soil Cd concentration in the study area was higher than that around Gudao Town, a typical oil-producing region of the Shengli Oilfield in the Yellow River Delta, China^12^, and from Yellow River Delta, a traditional oil field in China^9^, but was lower than that around two crude oil flow stations in the Niger Delta, Nigeria^36^. The concentrations of other PTEs were higher than the corresponding element concentrations, detected in the soil around Gudao Town, a typical oil-producing region of the Shengli Oilfield in the Yellow River Delta, China^12^, from Yellow River Delta, a traditional oil field in China^9^, and around two crude oil flow stations in the Niger Delta, Nigeria^36^. The above analysis exhibited that PTEs in the oil-affected soils had a certain degree of accumulation and may be affected by human activities.

**Table 1:** Statistical characteristics for potential toxic elements in in the study area (mg·kg^−1^).

	As	Cd	Cr	Cu	Mn	Ni	Pb	Zn	Ba
Minimum	17.42	0.06	231.15	22.72	260.53	103.13	289.12	55.65	204.52
Mean	22.55	0.32	1222.21	43.47	628.97	282.84	339.86	137.55	614.63
Maximum	39.19	0.78	6558.27	90.88	936.77	541.28	373.70	319.11	1040.48
Standard deviation	4.54	0.17	1726.00	15.99	134.70	104.45	23.64	77.18	206.10
Coefficient of variation	0.20	0.54	1.41	0.37	0.21	0.37	0.07	0.56	0.34
Background value	12.3	0.17	86	30.7	712	37.3	26.7	83.6	469
Risk screening value	20	20	–	2000	–	150	400	–	–
Grade II	40	0.30	150	50	–	40	250	200	–

### Levels of PTEs enrichment and pollution

**Figure 2:**
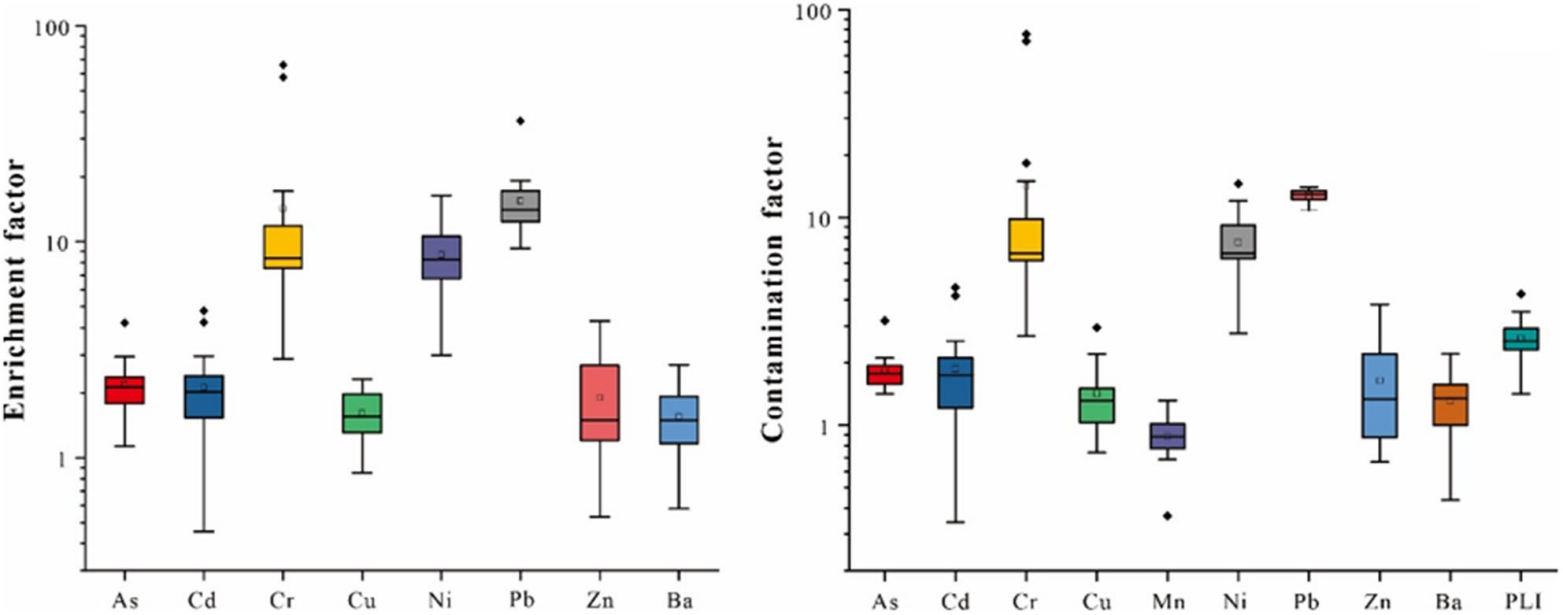
The map of enrichment factor and contamination factor of PTEs in the study area.

The EF and PLI of soil PTEs in the study area were calculated to evaluate the pollution degree of soil PTEs. The calculation results of EF and PLI were shown in Fig. 2 and Table S4. From Fig. 2, the mean EF values of PTEs were showed as Pb > Cr > Ni > As > Cd > Zn > Cu > Ba. The mean EFs of all PTEs were greater than 1. Among them, the average EF of Cu, Zn and Ba was between 1 and 2, which was slightly enriched. And As (2.18) and Cd (2.12) were moderately enriched. In particular, the average EF values of Cr, Ni and Pb were 14.23, 8.69 and 15.45, respectively, reaching a significant enrichment level, and all samples of Cr, Ni and Pb were at moderate or above enrichment, of which 10% of the Cr samples were extreme pollution, 85% of Cr samples, 95% of Ni and 5% of Pb (Table S4) were significantly enriched. These proved that these PTEs were generally enriched in the study area, especially Cr, Ni and Pb.

Except Mn, the average CF values of other PTEs were all > 1 (Fig. 2), indicating that the accumulation of Mn in the study area was relatively light, and there was no obvious Mn pollution. The CF values of all samples of As, Cr, Ni and Pb, 80% of Cd samples, 75% of Cu samples, 30% of Mn samples, 65% of Zn samples and 75% of Ba samples (Table S4) were higher than 1. And the mean CF values of Cr, Ni and Pb were 14.21, 7.58 and 12.73, respectively, certifying that the pollution of Cr, Ni and Pb in the study area was considerably serious. PLI was calculated based on the CF value of PTEs, and the results were shown in Fig. 2. The average value of PLI was 2.62, indicating that the soil PTEs in the study area were seriously polluted.

### Spatial distribution of soil PTEs in the study area

**Figure 3:**
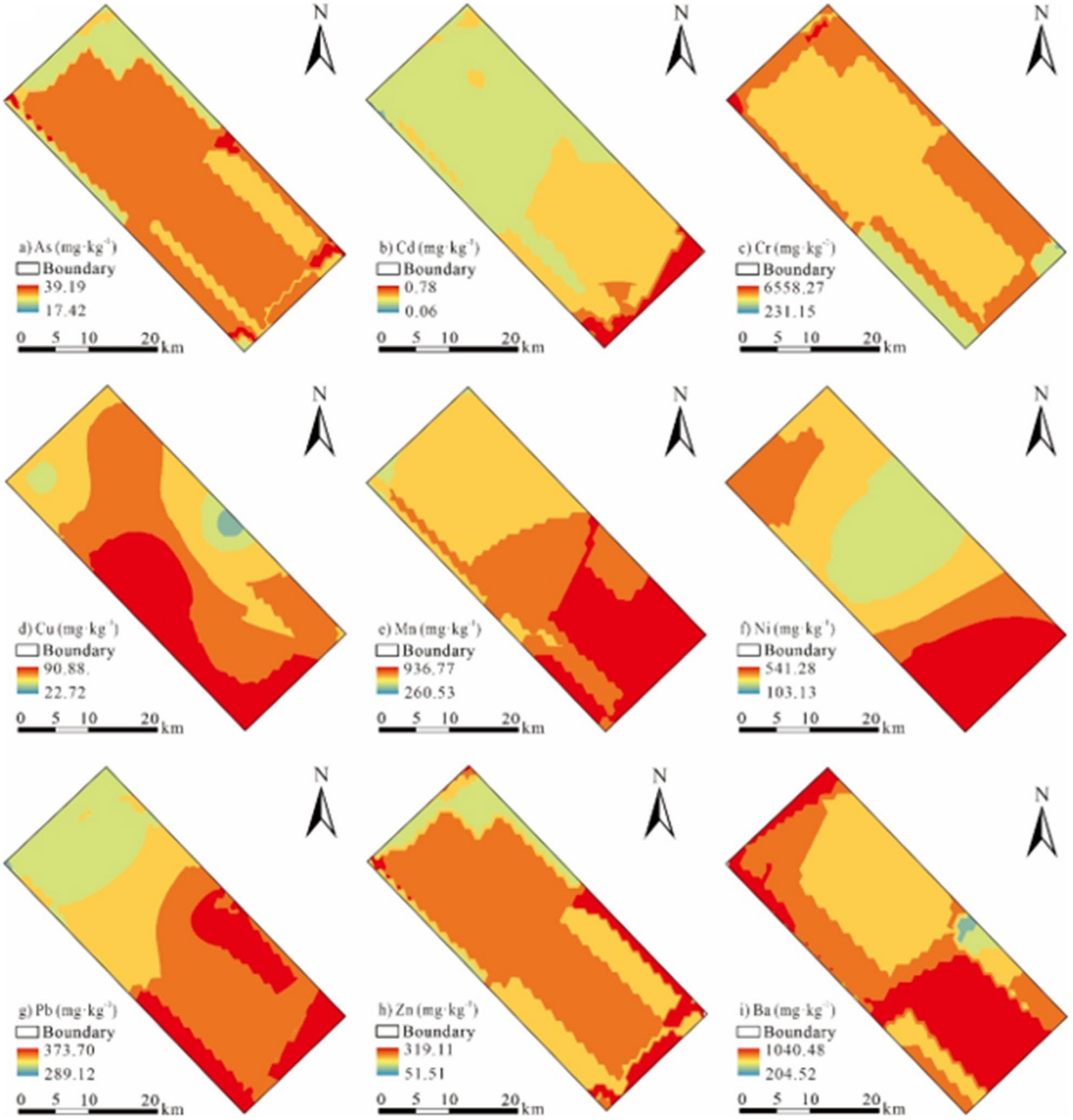
spatial distribution map of soil PTEs in the study area.

Geostatistical analysis was utilized to do ordinary Kriging interpolation of the PTEs in the study area, the results were shown in Fig. 3. As shown in Fig. 3, the spatial distribution of As, Cr, Ni, Zn and Ba was relatively consistent, and their hot spots were concentrated in the southeast, northwest, and central and eastern parts of the study area where oil wells were distributed. The spatial distribution of Cr and Ni exhibited that there were large-scale hotspots near the oil wells, and the content of Cr and Ni in these hotspots was much higher than second-level environmental quality standards of China, which proved that the content of soil Cr and Ni was significantly affected by the oil production activities of the oil production plant. There were crude oil leaks in B and C, and the contents of Zn and Ba in the vicinity of these two oil wells were relatively high, indicating that soil Zn and Ba in this area may be affected by the crude oil leakage, resulting in a certain degree of accumulation in the soil. The area with the second highest As content mainly resided in the middle of the study area. According to the survey, the herbicides were sprayed every year around the H oil well in the middle of the study area, indicating that the accumulation of As in the soil was not only related to oil extraction activities, but also to the use of pesticides (contains copper arsenate, sodium arsenate, etc.)^10, 14^. In addition, the hot spots of spatial distribution of Pb, Cd and Mn were concentrated in the southeast, and Cu was mainly concentrated in the southeast and midwest. As analyzed above, in addition to Mn, the PTEs Pb, Cd and Cu all have a certain degree of accumulation. And the investigation found that there were many petroleum machinery manufacturing plants in the central and eastern part of the study area, therefore, the accumulation of Pb, Cd and Cu in the soil may be related to factors such as petroleum extraction, crude oil leakage and machinery manufacturing. The above analysis indicated that the influence of human activities is evident on the distribution of soil PTEs^3, 23^.

### Potential ecological risk assessment

The potential ecological risk assessment model after adjusting the threshold was used to evaluate the PER of the oil production plant. The individual potential ecological risk of PTEs was shown in Table 2. From Table 2, the average ${E}_{r}^{i}$ values of PTEs were Cr > Pb > Cd > Ni > As > Cu > Zn > Mn. The average ${E}_{r}^{i}$ values of Cr and Pb were 79.62 and 63.64, respectively, reaching a relatively high level of potential ecological risk; the average ${E}_{r}^{i}$ values of Cd and Ni were 55.95 and 37.91, respectively, which were at medium potential ecological risk level; the average ${E}_{r}^{i}$ values of other PTEs were all lower than 30, with minor potential ecological risk. Specifically, all samples of Cu, Mn and Zn were at slight potential ecological risk level; 5% of As samples, 80% of Cd, 85% of Cr, 80% of Ni and 100% of Pb (Table S5) were at medium and above potential ecological risk. In particular, the potential ecological risks of 35% of Cd samples, 10% of Cr samples, 5% of Ni samples and 80% of Pb samples (Table S5) were relatively high, 10% Cd samples reached high potential ecological risk level, and 10% Cr samples had extremely high potential ecological risk. In summary, Geostatistical analysis shows that the hotspot distribution of all PTEs in the study area is almost related to the distribution of oil wells. In addition, the hotspot distribution of PTEs may also be related to factors such as agricultural and industrial activities^3^. The average value of PER in the study area was 265.08, and the proportions of the three risk levels of medium, slightly high and high were 5%, 75% and 20%, respectively (Table S5). It proved that the study area was at a higher potential ecological risk. Among them, the PER values of samples A, B, D, E, F, G, H, I and J (Table 2) were all greater than 280, reaching fairly high ecological risk.

**Table 2:** Single ecological risk index and potential ecological risk of soil PTEs in study area.

Sample	${E}_{r}^{i}$								PER
	As	Cd	Cr	Cu	Mn	Ni	Pb	Zn	
A(A1)	14.84	26.32	427.25	14.80	1.32	40.81	61.24	0.70	587.27
A(A2)	15.41	22.67	15.06	3.70	0.37	13.82	66.40	0.78	138.20
B(B1)	19.92	34.89	49.59	7.82	0.93	50.78	59.31	0.89	224.12
B(B2)	19.15	125.85	30.80	10.15	0.88	28.27	54.14	3.78	273.03
B(B3)	19.01	47.28	36.04	4.86	0.76	31.90	69.98	2.12	211.96
C	20.66	37.90	35.00	4.50	1.06	31.65	67.44	1.34	199.54
D(D1)	19.66	62.33	36.46	5.95	0.85	32.00	68.59	1.32	227.15
D(D2)	21.12	60.63	29.58	6.95	0.91	26.38	57.10	2.60	205.27
D(D3)	17.46	44.28	35.30	6.68	0.73	31.70	66.42	2.31	204.86
D(D4)	16.55	40.20	34.61	4.74	0.78	32.92	63.30	0.84	193.94
E(E1)	31.86	137.73	83.53	11.06	1.08	59.94	63.21	3.82	392.23
E(E2)	16.13	64.20	50.77	6.99	0.91	50.10	67.01	1.19	257.32
E(E3)	18.10	52.39	15.55	6.45	0.97	14.45	67.62	2.50	178.04
F(F1)	14.93	10.24	40.29	5.88	0.75	38.62	67.03	0.67	178.40
F(F2)	19.14	27.60	59.40	7.15	0.68	56.11	65.69	0.86	236.64
G(G1)	16.93	61.25	102.18	10.21	1.06	72.56	63.24	1.34	328.77
G(G2)	15.51	51.89	38.64	5.48	0.86	34.27	61.02	0.99	208.66
H	14.16	71.93	35.40	6.20	0.81	32.94	67.35	1.51	230.30
I	17.35	63.10	395.55	4.76	1.07	37.61	61.68	1.52	582.64
J	18.83	76.39	41.44	7.26	0.89	41.47	55.14	1.86	243.27
Mean	18.34	55.95	79.62	7.08	0.88	37.91	63.64	1.65	265.08

### Human health risk assessment

The non-carcinogenic risk assessment of As, Cd, Cr, Cu, Mn, Ni, Pb, Zn and Ba in the soils of the study area was carried out, and the assessment results were shown in Table 3. The THI values of children and adults under the three exposure routes of soil PTEs in the study area were 7.31 and 1.03, respectively, and the THI values were all > 1, which indicated that soil PTEs around the oil production plants posed significant non-carcinogenic health risks to children and adults. The non-carcinogenic hazardous quotient (HQ) of children and adults in Table 3 revealed that the HQ of all PTEs for adults under each exposure route was less than 1, while the HQ of Cr and Pb for children under the oral intake route was greater than 1, which were 4.91 and 1.17, respectively. For HQ with different exposure routes of the same PTE, each soil PTE presented the risk of oral ingestion > oral and nasal inhalation risk > skin contact risk. The result was in agreement with the reports^14, 37^. Therefore, oral intake was the main exposure route of non-carcinogenic risk, and oral intake of Cr and Pb caused serious non-carcinogenic risk to children. Statistical analysis of HI for soil PTEs in the study area showed that the HI values of PTEs for children were significantly higher than those of adults, and the HI values of PTEs in children and adults were all Cr > Pb >  > As > Ni > Mn > Ba > Cu > Zn > Cd. Among them, the HI values of all PTEs for adults were less than 1, indicating that the non-carcinogenic risks caused by a single PTE did not have a significant impact on adults; while the HI values of Cr and Pb for children were 4.93 and 1.17 greater than 1, indicating that they have caused serious non-carcinogenic risk to local children. In addition, the HI values of As and Ni for children and the HI values of As, Cr and Pb for adults were all greater than 0.1, which requires attention. In summary, children suffered from significant non-carcinogenic risk, and adults suffered from minor non-carcinogenic risk in the study area; soil Cr and Pb were the most important non-carcinogenic risk factors for children and adults in the study area.

**Table 3:** Non-cancer and cancer risk assessment of adults and children under different exposure routes.

	Oral ingestion		Respiratory inhalation		Dermal contact			
	Children	Adult	Children	Adult	Children	Adult	Children	Adult
**Hazard quotient and hazard index of each PTE**							**HI**	
As	9.07E−01	1.27E−01	2.50E−05	1.40E−05	6.98E−08	1.64E−08	9.07E−01	1.27E−01
Cd	3.82E−03	5.35E−04	1.05E−05	5.90E−06	2.86E−06	6.74E−07	3.84E−03	5.42E−04
Cr	4.91E+00	6.88E−01	1.42E−02	7.96E−03	1.43E−06	3.37E−07	4.93E+00	6.96E−01
Cu	2.62E−02	3.67E−03	7.02E−07	3.93E−07	1.06E−07	2.49E−08	2.62E−02	3.67E−03
Mn	5.42E−02	7.58E−03	4.18E−03	2.34E−03	2.18E−06	5.13E−07	5.84E−02	9.93E−03
Ni	1.71E−01	2.39E−02	4.57E−06	2.56E−06	1.06E−07	2.49E−08	1.71E−01	2.39E−02
Pb	1.17E+00	1.64E−01	3.23E−05	1.81E−05	1.91E−07	4.49E−08	1.17E+00	1.64E−01
Zn	5.53E−03	7.74E−04	1.52E−07	8.54E−08	1.43E−07	3.37E−08	5.53E−03	7.74E−04
Ba	3.71E−02	5.19E−03	1.02E−06	5.72E−07	2.86E−08	6.74E−09	3.71E−02	5.19E−03
THI	–	–	–	–	–	–	7.31E+00	1.03E+00
**Cancer risk of each PTE**							**CR**	
As	3.50E−05	2.12E−05	9.71E−09	2.36E−08	2.69E-12	2.75E-12	3.50E−05	2.12E−05
Cd	1.67E−07	1.01E−07	5.69E-11	1.38E-10	4.91E-11	5.00E-11	1.67E−07	1.02E−07
Cr	6.32E−04	3.83E−04	1.46E−06	3.55E−06	1.47E-10	1.50E-10	6.33E−04	3.87E−04
Ni	2.46E−04	1.49E−04	–	–	–	–	2.46E−04	1.49E−04
Pb	2.99E−05	1.81E−05	–	–	–	–	2.99E−05	1.81E−05
TCRI	–	–	–	–	–	–	9.44E−04	5.75E−04

In this study, soil As, Cd, Cr, Ni and Pb from the study area were assessed for carcinogenic risk, and the results were shown in Table 3. The TCRI of children and adults under the three exposure routes of these five PTEs were 9.44E−04 and 5.75E−04, respectively, indicating that soil PTEs around the oil production plants have caused serious carcinogenic risk to local children and adults. The CR values of children and adults showed that the CR values of Cr (6.33E−04) and Ni (2.64E−04) for children, and Cr (3.87E−04) and Ni (1.49E−04) for adults were all greater than 10^–4^. In addition, As, Cr and Cd all presented oral intake risk > oronasal inhalation risk > skin contact risk. In conclusion, Cr and Ni caused serious carcinogenic risk for children and adults in the study area, and oral intake was also the primary way of carcinogenic risk. The CRI statistics of adults and children exhibited that the CRI values of all PTEs were lower than those of children. The CRI values of the PTEs in adults and children under the three exposure routes were Cr > Ni >  > As > Pb >  > Cd. Among them, the CRI values of Cr and Ni in children and adults by oral intake were both greater than 10^–4^, showing a strong carcinogenic risk. It is noteworthy that the assessment based on total concentrations of PTEs in soil might overestimate potential health risks^38^. The above analysis revealed that both children and adults in the study area suffered from serious carcinogenic risks, and Cr and Ni were the chiefly carcinogenic risk factors.

## Conclusions

In this research, the contents of As, Cd, Cr, Cu, Ni, Pb, Zn and Ba all exceeded their background values. And compared with the control area, PTEs in the soil of the study area had a significant accumulation. The results of EF and PLI showed that the mean EFs of all PTEs were greater than 1, and in particular, Cr (14.23), Ni (8.69) and Pb (15.45) reached a significant enrichment level; the average value of PLI was 2.62, and the soil PTEs in the study area were seriously polluted. Geostatistical analysis revealed that the hotspot distribution of all PTEs in the study area was almost related to the distribution of oil wells. In addition, the hotspot distribution of PTEs may also be related to factors such as agricultural and industrial activities. The average PER in the study area was 265.08, reaching a higher potential ecological risk. Among them, the proportions of the three risk levels of medium, slightly high and high were 5%, 75% and 20%, respectively. The health risk assessment of adults and children in the study area showed that the THI values of children and adults were 7.31 and 1.03, respectively, indicating that children suffered from significant non-carcinogenic risk, and adults suffered from minor non-carcinogenic risk. Besides, soil Cr and Pb were the most important non-carcinogenic risk elements. The TCRI of children and adults under the three exposure routes of these five PTEs were 9.44E−04 and 5.75E−04, respectively, indicating that soil PTEs around the oil production plant have caused serious carcinogenic risk to local children and adults. Cr and Ni were the chiefly carcinogenic risk factors. In addition, whether it was carcinogenic risk or non-carcinogenic risk, oral ingestion was the primary way to cause health risks.

## Supplementary Information

Supplementary Tables.

## Data Availability

Some or all data, models, or code generated or used during the study are available from the corresponding author by request.
