# Supplementary material for: Ecological risk and health risk analysis of soil potentially toxic elements from oil production plants in central China
Source: Sci Rep. 2022 Oct 12;12:17077. doi: 10.1038/s41598-022-21629-y (PMC9556517; doi:10.1038/s41598-022-21629-y)
Supplement: Supplementary file 1 — Supplementary Tables. [file 41598_2022_21629_MOESM1_ESM.docx]

Table S1

Hakanson grading standard and the improved grading standard of this research.

| Level | Hakanson grading standard ^[1]^ | | Classification standard of this study | |
| --- | --- | --- | --- | --- |
|  | $E_{r}^{i}$ | PER | $E_{r}^{i}$ | PER |
| 1（Slight） | <40 | <150 | <30 | <70 |
| 2（medium） | 40 – 80 | 150 – 300 | 30 – 60 | 70 – 140 |
| 3（Higher） | 80 – 160 | 300 – 600 | 60 – 120 | 140 – 280 |
| 4（High） | 160 – 320 | > 600 | 120 –240 | >280 |
| 5（Extremely high） | >320 | — | >240 | — |

[1] Hakanson L. (1980). An ecological risk index for aquatic pollution control. a sedimentological approach. *Water Research.* 14(8): 975–1001.

Table S2

Parameters and their definitions, values in Eq (6) - Eq (8).

| Parameter | Definition | | Value | | | | References |
| --- | --- | --- | --- | --- | --- | --- | --- |
|  |  |  | Children | | Adults | |  |
| C (mg·kg^-1^) | Contents of PTEs |  | |  | | This study | |
| IngR (mg·kg^-1^) | Ingestion rate | 200 | | 100 | | US EPA, 2011 | |
| EF (days·year^-1^) | Exposure frequency | | 350 | | 350 | | US EPA, 2011 |
| ED (years) | Exposure duration | | 6 | | 26 | | US EPA, 2011 |
| BW (kg) | Body weight | | 15.9 | | 56.8 | | CNEPM, 2014 |
| AT (day) | Averaging time | | Non – Cancer: ED×365 Cancer: 70 ×365 | | | | US EPA, 2011 |
| InhR (m^3^·day^-1^) | Inhalation rate | | 7.5 | | 15 | | US EPA, 2011 |
| PEF (m^3^·kg^-1^) | Particle emission factor | | 1.36×10^9^ | | 1.36×10^9^ | | US EPA, 2011 |
| SA (cm^2^) | Exposed skin area | | 2373 | | 5700 | | US EPA, 2011 |
| AF (mg·(cm^2^·day)^-1^) | Adsorption coefficient | | 0.2 | | 0.07 | | US EPA, 2011 |
| ABS | Dermal absorption factor | | 0.001 | | 0.001 | | US EPA, 2011 |

CNEPM (China National Environmental Protection Ministry), 2014. Technical Guidelines for Risk Assessment of Contaminated Sites. China Environmental Science Press, Beijing. HJ25.3-2014. (In Chinese).

US EPA, 2011. Exposure factors handbook. National Center for Environmental Assessment. Office of Research and Development, Washington, D.C.

Table S3 Reference dose of non-carcinogenic PTEs and slope factor of carcinogenic PTEs.

| PTEs | RfD/mg·(kg·d)^-1^ | | | SF/(kg·d)·mg^-1^ | | | References |
| --- | --- | --- | --- | --- | --- | --- | --- |
|  | Oral ingestion | Respiratory inhalation | Dermal contact | Oral ingestion | Respiratory inhalation | Dermal contact |  |
| As | 3.00E-04 | 3.00E-04 | 1.23E-04 | 1.50E+00 | 1.51E+01 | 3.66E+00 | US EPA, 2011 |
| Cd | 1.00E-03 | 1.00E-05 | 1.00E-05 | 5.10E-01 | 6.30E+00 | 2.00E+01 | US EPA, 2011 |
| Cr | 3.00E-03 | 2.86E-05 | 6.00E-05 | 5.00E-01 | 4.20E+01 | 2.00E+01 | US EPA, 2011 |
| Cu | 2.00E-02 | 2.06E-02 | 5.40E-03 | - | - | - | US EPA, 2011 |
| Mn | 1.40E-01 | 5.00E-05 | 1.84E-03 | - | - | - | US EPA, 2011 |
| Ni | 2.00E-02 | 2.06E-02 | 5.40E-03 | 8.40E-01 | - | - | US EPA, 2011 |
| Pb | 3.50E-03 | 3.50E-03 | 5.25E-04 | 8.50E-02 | - | - | US EPA, 2011 |
| Zn | 3.00E-01 | 3.00E-01 | 6.00E-02 | - | - | - | US EPA, 2011 |
| Ba | 2.00E-01 | 2.00E-01 | 2.00E-01 | - | - | - | US EPA, 2011 |

US EPA, 2011. Exposure factors handbook. National Center for Environmental Assessment. Office of Research and Development, Washington, D.C.

Table S4

The feature of contamination factor (CF) and enrichment factor (EF) values.

|  | | As | Cd | Cr | Cu | Mn | Ni | Pb | Zn | Ba |
| --- | --- | --- | --- | --- | --- | --- | --- | --- | --- | --- |
| CF | Minimum | 1.42 | 0.34 | 2.69 | 0.74 | 0.37 | 2.76 | 10.83 | 0.67 | 0.44 |
|  | Mean | 1.83 | 1.87 | 14.21 | 1.42 | 0.88 | 7.58 | 12.73 | 1.65 | 1.31 |
|  | Maximum | 3.19 | 4.59 | 76.26 | 2.96 | 1.32 | 14.51 | 14.00 | 3.82 | 2.22 |
|  | CF>1 (%) | 100 | 80 | 100 | 75 | 30 | 100 | 100 | 65 | 75 |
| EF | Minimum | 1.13 | 0.45 | 2.87 | 0.85 | - | 2.99 | 9.31 | 0.53 | 0.58 |
|  | Mean | 2.18 | 2.12 | 14.23 | 1.61 | - | 8.69 | 15.45 | 1.90 | 1.55 |
|  | Maximum | 4.21 | 4.77 | 66.15 | 2.31 | - | 16.39 | 36.29 | 4.30 | 2.70 |
|  | EF<1(%) | - | 10 | - | 10 | - | - | - | 15 | 25 |
|  | 1<EF<2(%) | 50 | 35 | - | 65 | - | - | - | 45 | 55 |
|  | 2<EF<5(%) | 50 | 55 | 5 | 25 | - | 5 | 95 | 40 | 20 |
|  | 5<EF<20(%) | - | - | 85 | - | - | 95 | 5 | - | - |
|  | 20<EF<40(%) | - | - | - | - | - | - | - | - | - |
|  | EF>40(%) | - | - | 10 | - | - | - | - | - | - |

Table S5

Percentage of individual ecological risks and comprehensive potential ecological risks of soil PTEs in the study area.

|  | Percentage of ecological risks at all levels (%) | | | | |
| --- | --- | --- | --- | --- | --- |
|  | <30 | 30-60 | 60-120 | 120-240 | >240 |
| As | 95 | 5 | - | - | - |
| Cd | 20 | 35 | 35 | 10 | - |
| Cr | 15 | 65 | 10 | - | 10 |
| Cu | 100 | - | - | - | - |
| Mn | 100 | - | - | - | - |
| Ni | 20 | 75 | 5 | - | - |
| Pb | - | 20 | 80 | - | - |
| Zn | 100 | - | - | - | - |
|  | <70 | 70-140 | 140-280 | >280 |  |
| PER | - | 5 | 75 | 20 |  |
